# Neurobiological Mechanisms of Acupuncture 2014

**DOI:** 10.1155/2014/765068

**Published:** 2014-11-27

**Authors:** Lijun Bai, Richard E. Harris, Jian Kong, Lixing Lao, Vitaly Napadow, Baixiao Zhao

**Affiliations:** ^1^The Key Laboratory of Biomedical Information Engineering, Ministry of Education, Department of Biomedical Engineering, School of Life Science and Technology, Xi'an Jiaotong University, Xi'an 710049, China; ^2^Department of Anesthesiology, University of Michigan, Ann Arbor, MI 48109, USA; ^3^Department of Psychiatry, Massachusetts General Hospital (MGH), Harvard Medical School, Boston, MA 02129, USA; ^4^School of Chinese Medicine, The University of Hong Kong, 10 Sassoon Road, Pokfulam, Hong Kong; ^5^Center for Integrative Medicine (CIM), School of Medicine, University of Maryland Baltimore (UMB), Baltimore, MD 21201, USA; ^6^Martinos Center for Biomedical Imaging, Massachusetts General Hospital, Harvard Medical School, Boston, MA 02115, USA; ^7^School of Acupuncture-Moxibustion and Tuina, Beijing University of Chinese Medicine, Beijing 100029, China

Acupuncture is currently gaining popularity as an important modality of complementary and alternative medicine (CAM) in the western world. Acupuncture has shown efficacy in the treatment of postoperative and chemotherapy nausea and vomiting. It has also become a beneficial adjunct for pain management, stroke rehabilitation, and depression. Partly as a consequence of its public acceptance, increasing attention is being paid to explore the scientific explanation regarding the physiological mechanism of acupuncture. This special issue on neurobiological mechanisms of acupuncture compiled 9 articles, most of which represent novel primary research and explore the neurophysiologic mechanisms of acupuncture contributing to current hypotheses of acupuncture action.

For instance, stroke is responsible for increasingly high rates of mortality and disability. Acupuncture treatments for improved motor performance following stroke may lead to a remodeling of the neural network architecture of the entire motor system towards a more physiological state. Dr. Z. Xie et al. indicated that acupuncture can enhance bidirectional effective connectivity between the cerebellum and primary sensorimotor cortex, which contributed to the improvement of movement coordination and motor learning in the subacute stroke patients. This study demonstrated additional acupuncture mechanisms supporting stroke rehabilitation, which expand the scope of the study population and relay additional longitudinal observations focusing on the relationships between different brain-based variables and the degree of clinical recovery after acupuncture.

Two papers focus on potential neural mechanisms underlying acupuncture treatment for insomnia and peripheral facial nerve palsy. Acupuncture is widely used in insomnia clinically and empirically; however, the potential neural mechanism underlying the therapeutic effects of acupuncture is still unknown. Acute sleep loss, or sleep deprivation (SD), to some extent, is an alternative form of acute insomnia. Dr. L. Gao et al. investigated the activation patterns of acupuncture in SP6 under different sleep conditions, that is, normal sleep and after a night of total SD. They found that acupuncture at SP6 can induce more widespread and significant brain activations in the SD condition, compared with that of normal sleep. The salience brain network, which also processes interoceptive and autonomic information, may partly underpin the mechanism underlying acupuncture in the restoration of sleep deprivation. Dr. H. Tang et al. investigate the effects of electroacupuncture on the alleviation of peripheral facial nerve palsy (PFN) symptoms induced by herpes simplex virus type 1 (HSV-1) infection. Facial nerve function recovered more quickly in the electroacupuncture group, which was significantly lower in HSV-1 DNA quantity at day 3 and day 7, compared to the animal control group. Electroacupuncture alleviated symptoms, facilitated affected nerve recovery, and promoted the reduction of HSV-1. The authors suggested that further study is needed to elucidate the precise mechanism of acupuncture in reducing HSV-1.

Manual acupuncture (MA) mainly contained monotype manipulations and multitype manipulations with different stimulation parameters (frequency, angle and depth, etc.). Dr. S. Hong et al. found that MA with different frequencies can induce distinct changes of the firing rate of excitatory gastric-related wide dynamic range (WDR) neurons in spinal dorsal horn (SDH) in normal rats following graded acute gastric distension (GD). Dr. L. Yu et al. further explored the different role of wide dynamic range (WDR) and subnucleus reticularis dorsalis (SRD) neurons in response to acupuncture at different acupoints. They suggested that the function of viscerosomatic convergence-facilitation at the spinal and medulla levels may be related to an acupoint sensitization phenomenon.

It is not known which aspects of the acupuncture treatment, such as the mode of stimulation or location of acupuncture points, are specific to produce different physiological effects. Dr. Y. Shan et al. aimed to evaluate the functional specificity of the Siguan acupoint, a combination of Hegu (LI4) and Taichong (LR3), and used a sham point as control. They found that real acupuncture can induce more increased activity in the somatosensory cortex, limbic-paralimbic system, and basal ganglia, compared with the sham control. They also found that brain activity induced by multiacupoint acupuncture correlates closely with that following individual acupoint stimulation, which has important implications for interpretation of the many previous single acupoint stimulation studies. Dr. C. Wu et al. investigated the changes in amplitude of low-frequency fluctuation and regional homogeneity in the brain fMRI signal induced by acupuncture at Taichong (LR3) acupoint. They found that acupuncture at LR3 can specifically activate or deactivate brain areas related to vision, movement, sensation, emotion, and analgesia, while sham acupuncture has a certain effect on psychological processes and does not affect brain areas related to function.

By gathering these papers, we hope to enrich our readers with respect to the underlying neurobiological mechanisms of acupuncture and consider important research questions for future studies, such as expanding research through larger samples, building clinical relevance into the design of basic research, and developing reliable biomarkers (i.e., neuroimaging) and clinical outcome measures of physiological responses to needling in humans and animals.

*Lijun Bai* *Lijun Bai* *Richard E. Harris* *Richard E. Harris* *Jian Kong* *Jian Kong* *Lixing Lao* *Lixing Lao* *Vitaly Napadow* *Vitaly Napadow* *Baixiao Zhao* *Baixiao Zhao*

